# Probabilistic Record Linkage (PRL) advances between Surveillance Data and HES datasets using the Expectation-Maximization (EM) algorithm

**DOI:** 10.23889/ijpds.v1i1.371

**Published:** 2017-04-19

**Authors:** Nikolaos Panagiotopoulos, Mehdi Minaji, Richard Pebody

**Affiliations:** 1 Public Health England

## Objectives

This study shows how to improve PRL when using a limited number of personal identifiable information (PIIs) available in HES such as NHS number, DOB, Hospital Number, Gender and Postcode. The focus is on new approaches in categorisation of m-u probabilities for fields related to Postcode. In addition, a brief outline of our procedure to generate an appropriate training dataset will be presented as well as reasons for splitting HES dataset into single and multi-admissions, and proposed techniques to avoid computational cost.

## Approach

PIIs such as DOB and Postcode can be categorised in order to increase the number of potential comparison vectors which is necessary in order to deal with the multi-million size of the HES dataset. We propose a multinomial approach for comparison between postcodes both for agreement and disagreement case scenarios, based on geographical information and a similarity score resulting from a Probit model. In the absence of a `gold-standard' dataset, generating an appropriate training set is possible by combining two-way Deterministic Linkage (DL) and simple random sampling. Splitting HES into single and multi-admissions is recommended for reasons related to computational performance and demographic differences of the underlying population. Selection of the lower and upper thresholds regarding linkage weights can be done in a practical way by using conditional Normal mixture modelling, or more formally by using sampling techniques based on Fellegi-Sunter decision rule. We show how the latter can be further improved avoiding the computational cost by introducing appropriate mapping functions.

## Results

We show the advantages of PRL over DL between Surveillance Laboratory Flu Data and HES. Preliminary results indicate an additional 10% of matches for < 1% expected false positives. EM convergence problems when treating missing values as separate outcome category will be discussed comparing results with the suggested re-normalisation approach in the bibliography.

## Conclusion

PRL between Laboratory Surveillance Data and Hospital Episode Statistics (HES) is a common practice and performed in a number of epidemiological studies conducted at Public Health England (PHE). Therefore, improving linkage will subsequently benefit those studies. Furthermore, this study proposes new approaches for Postcode comparison, and the utilisation of mapping functions to avoid the computational cost of the Fellegi-Sunter decision rule. These approaches could be used in a wide range of applications. Finally, we suggest a structural way of handling the HES dataset in order to achieve high efficiency and robustness of linkage.

